# Provision of E-Cigarettes for Smoking Cessation in Pregnancy: Perceptions and Experiences of Pregnant Women from Two UK Sites

**DOI:** 10.3390/ijerph21040472

**Published:** 2024-04-12

**Authors:** Eleanor Lutman-White, Riya Patel, Lauren Bell, Deborah Lycett, Kelly Hayward, Ruth Sampson, Janani Arulrajah, Maxine Whelan

**Affiliations:** 1 Centre for Healthcare and Communities, Coventry University, Coventry CV1 5FB, UK; ad6406@coventry.ac.uk (E.L.-W.); lauren.bell@coventry.gov.uk (L.B.); ab5042@coventry.ac.uk (D.L.); 2 Centre for Ethnic Health Research, NIHR Applied Research Collaboration-East Midlands (ARC-EM), University of Leicester, Leicester LE1 7RH, UK; rp526@leicester.ac.uk; 3 Warwickshire Public Health Team, Warwick CV34 4UL, UK; 4 Bath and North Somerset Public Health Team, Bristol BS31 1FS, UK

**Keywords:** smoking cessation, e-cigarettes, pregnancy, qualitative

## Abstract

Introduction: Smoking in pregnancy is associated with negative health outcomes for both mothers and babies; e-cigarettes, which contain nicotine without hazardous tobacco, may offer an additional smoking cessation strategy for pregnant women. Although e-cigarettes are being increasingly offered within services, there is limited understanding about whether e-cigarettes can improve smoking cessation support for pregnant individuals. This study aimed to explore service users’ experiences of using e-cigarettes as a tool for smoking cessation during pregnancy. Methods: Semi-structured interviews were conducted with 14 women who had accepted one of two pilots and were analysed using inductive reflexive thematic analysis. The findings from each site were integrated to develop qualitative insight. Results: Participants largely had positive perceptions of the free and easy-to-use e-cigarette, preferring it to nicotine replacement therapies. The desire to have a healthy pregnancy and baby and the inclusion of non-judgemental behavioural support facilitated motivation to quit. Many participants reduced or quit tobacco use, with positive social and health implications reported. However, numerous barriers to quitting were present and intentions about long-term quitting of combustible cigarettes and e-cigarettes were mixed and uncertain. Conclusions: Providing e-cigarettes within smoking cessation services was indicated to be a positive and effective strategy for pregnant women trying to quit tobacco. However, numerous barriers to quitting and staying quit remained, suggesting scope for further improvements to smoking cessation support for pregnant women.

## 1. Introduction

There are many recognised detrimental health effects associated with smoking tobacco during pregnancy, including preterm birth, foetal mortality, stillbirth, birth malformations (e.g., oral clefts), low birth weight, and sudden infant death syndrome [1]. Women who smoke during pregnancy may also experience negative moral judgements and stigma [2] and are more likely to have poorer mental wellbeing that can further impede smoking cessation [3]. In the United Kingdom (UK), 8.8% of women are currently smoking at the time of birth [4], with national targets to reduce this figure to 6% [5]. With around 600,000 live births in England and Wales in 2022 [6], there is an urgent need to improve uptake and engagement for smoking cessation programmes targeting pregnant women. 

Nicotine replacement therapies (NRT), which include the addictive nicotine component without the hazards associated with tobacco, in forms such as patches, gum, and tablets [7], are often offered to support smoking cessation. Whilst NRT are indicated to be substantially safer than smoking cigarettes [8], to date, there is low-quality evidence investigating the efficacy of NRT for smoking cessation in pregnant populations and low adherence in trials [9,10]. NRT are recommended and endorsed as part of standard practice in the UK. However, pregnant women have identified the need for greater options and support for smoking cessation strategies [11]. Rates of smoking in pregnancy are also higher among people with more socioeconomic disadvantage [12], yet evidence suggests that women from lower socioeconomic backgrounds might benefit less from interventions currently available [13]. 

One alternative additional offer for smoking cessation in pregnant women is the use of e-cigarettes. E-cigarettes, also known as vapes, normally contain nicotine (in varying strengths) but do not contain the hazardous chemicals and tar inhaled via smoking tobacco [14]. Whilst the evidence base for the safety of e-cigarettes in pregnancy is still developing, they are predicted to have benefits, in terms of being a safer alternative to smoking; vaping products are viewed as substantially less harmful than smoking [15]. Growing in popularity, e-cigarette use has been found to be more effective than traditional NRT for smoking cessation in non-pregnant people [16,17], as well as more cost-effective to the National Health Service (NHS) [18]. Among pregnant women, evidence of the effectiveness of e-cigarettes is lacking [13,19]. A recent RCT has found that, after excluding participants who used non-allocated products, e-cigarettes were more effective in prolonged validated quit rates compared to the nicotine patch arm (6.8% vs. 3.6%) [20]. The authors described a similar safety profile for e-cigarettes as nicotine patches in pregnant women (including maternal outcomes, birth outcomes, and adverse events). Low birth weight was significantly less often reported in the arm using e-cigarettes. A secondary analysis of that RCT showed that regular use of e-cigarettes does not appear to be associated with adverse outcomes such as birth weight [21]. The evidence is still emerging about the use of e-cigarettes as a smoking harm reduction tool in pregnant women. There is geographical variation in how local governments and health trusts provide smoking cessation services to pregnant groups and only 11% of local governments currently offer e-cigarettes [22]. The provision of e-cigarettes as a smoking cessation strategy in pregnancy is increasing and considerable further evaluation is needed to understand whether e-cigarettes can offer improved smoking cessation support [23,24]. Specifically, more qualitative evaluation has been instructed to capture the experiences of pregnant individuals offered e-cigarettes as a smoking cessation and harm reduction tool [15,19,25]. To fill this knowledge gap, we explored the views of pregnant individuals using e-cigarettes as an alternative offer within smoking cessation services in two UK-based pilots.

## 2. Methods

### 2.1. Study Design

A qualitative evaluation in two UK sites (Warwickshire and Bath and Northeast Somerset) was conducted to explore service users’ experiences of a pilot to offer e-cigarettes as a tool for smoking cessation during pregnancy.

### 2.2. Context

The pilot has been described in depth elsewhere [26]. Implemented by two local authorities, e-cigarettes were added as an alternative tool within smoking cessation in pregnancy services with the aim of improving service engagement and outcomes. All women were referred to a specialist stop smoking in pregnancy service and offered e-cigarettes in addition to standard treatment (NRT and behavioural support) to help abstain from smoking. This was a 12-week programme with ongoing support throughout pregnancy and postnatally (as needed). In Pilot 1, women were supplied with up to two disposable e-cigarette devices a week with different available flavours, depending on smoking habits and verified by CO (carbon monoxide) readings. Each e-cigarette device provided 320 × 2 s puffs (roughly equating to 30–35 cigarettes, 18 mg of nicotine). The three flavours on offer were tobacco, menthol, and blueberry, with tobacco flavour encouraged at the beginning of a quit and fruity flavours later. In Pilot 2, pregnant women received a refillable and rechargeable pen-style e-cigarette with a choice of 10 e-liquid flavours (tobacco, menthol, and berry proving most popular) and nicotine strengths and provided CO readings in line with standard practice [27]. Both pilots offered service users information about e-cigarettes and only declared their use as a short-term harm reduction tool to support pregnant women to fully quit and that long-term it would be best for women and babies to not use any kind of cigarette.

### 2.3. Participant Selection: Sampling

Participants were women aged 18 years or older who had enrolled on the e-cigarette pilot at two sites. Pregnant women were not invited to interview if they had experienced pregnancy complications or a miscarriage.

### 2.4. Setting

Interviews were conducted either in person (n = 3, all for Pilot 1) or by telephone (n = 11) and participants were offered evening and weekend slots.

## 3. Participant Selection: Method of Approach

Stop smoking in pregnancy advisers (hereon referred to as ‘advisers’) held primary responsibility for participant recruitment. Advisers shared information about the interviews, with informed consent collected via paper or online (via Qualtrics). Individuals provided contact details and basic demographic information and agreed to be contacted by the research team. The research team arranged convenient days and times for the interviews. 

### 3.1. Data Collection

A semi-structured interview guide was developed with colleagues from the University of Bath and used for Pilot 1. The guide was adapted by Coventry University to use for Pilot 2 participants to ensure relevancy to Pilot 2. Interviews were conducted with as many service users as possible during the recruitment period (Pilot 1 ran from 18 October–19 September; Pilot 2 ran 22 July–23 May with a 3-month suspension from 22 October–22 December due to insufficient resources). No interviews were repeated. Interviews were transcribed manually, with all identifiable information deleted. The research team checked transcripts for accuracy against the recordings. Transcripts were not returned to participants.

### 3.2. Research Team and Reflexivity

In Pilot 1, interviews were conducted by one female researcher (RS) who had an MSc and 16+ years of local authority public health experience, specialising in tobacco control. In Pilot 2, two female researchers conducted the interviews (ELW, LT). Both authors had prior experience in interviewing and had obtained (ELW) or submitted (LT) PhDs at the point of data collection. ELW, RS, and JA had experience of being pregnant and receiving NHS maternity care. None of the interviewers were current smokers and one had previously smoked.

### 3.3. Data Analysis 

Transcripts were analysed using a reflexive thematic analysis approach [28]. After familiarisation via conducting and/or reading the interviews, transcripts were inductively coded (ELW) for comments, feelings, and experiences that related to the use of e-cigarettes within the pilot at a semantic level. Coded data were organised into appropriate sub-themes and then themes, using an Excel workbook for each site. Data for Pilot 1 and Pilot 2 were initially analysed separately and then combined, merging and revising sub-themes and themes. Theme development was discussed and revised (ELW, RP, LB, MW, and RS). Findings were presented and supported by anonymised data extracts from participants, chosen to reflect diverse participants and experiences. Verbatim quotes were modified to aid readability (i.e., removing word repetitions and hesitations). Quotes were indicated with the site and participant number (e.g., ‘P1b’ refers to pilot 1’s second interviewee).

## 4. Results

### 4.1. Participants

A more comprehensive outline of pilot uptake, engagement, and retention is presented elsewhere (Lutman-White et al.; under review). To summarise, 99 pregnant women accepted the service in Pilot 1 and 25 pregnant women accepted the e-cigarette in the pregnancy service in Pilot 2. Of these, 14 women (eight in Pilot 1 and six in Pilot 2) took part in interviews lasting 33 min on average, ranging from 19–76 min. Reasons for the poor uptake of interviews in Pilot 2 included difficulties in contacting people, withdrawal of consent, general complexity in people’s lives, and COVID-19 lockdowns. All interviewees reported a white ethnicity and on average were aged 27 years (ranging from 18–36 years). Prior to enrolling in the pilot, women primarily used rolling tobacco only (n = 6), cigarettes only (n = 4), cigarettes and rolling tobacco (n = 3), or were using cigarettes and e-cigarettes (n = 1). At the time of the interview, 9 participants had quit (6 from Pilot 1 and 3 from Pilot 2), 3 were dual using combustible cigarettes and an e-cigarette (1 from Pilot 1, 2 from Pilot 2), 1 had not quit (Pilot 2), and the outcome of the pilot was unknown for 1 participant (Pilot 1). 

### 4.2. Qualitative Findings

Thematic analysis generated three main themes, which were accompanied by quotes in Table 1.

#### 4.2.1. Theme 1: Perceptions of Key Aspects of the e-Cigarette Pilot

This theme reports participants’ perceptions of the e-cigarettes offered within the pilots (subtheme 1A), experiences of learning and using the e-cigarette (subtheme 1B), the role of behavioural support offered (subtheme 1C), and overall pilot perceptions (subtheme 1D).

##### Subtheme 1A: The E-Cigarette Offered

Most participants felt that the e-cigarettes offered through the pilots were easy to use, high quality, and compact and, where participants recalled their previous attempts with e-cigarettes, the pilot e-cigarette was preferred. Although pocket-sized, the need for careful transportation was noted. A few criticisms were related to the transparent and luminous design of one e-cigarette, with two Pilot 1 participants comparing it to a glow stick.

Individuals had mixed views on the types and nicotine strengths of e-cigarette or e-liquid flavours available. Some participants preferred a flavour that more closely replicated tobacco. Others chose a flavour that contrasted with tobacco or changed flavours over time; however, some participants struggled to find a flavour that they liked due to the preponderance of sweet flavours. To overcome this issue, a few participants suggested sampling flavours could help.

The e-cigarette being free was viewed positively by participants and was assumed to have increased access to those with a lower income. One participant reported that, due to financial constraints, they were therefore able to start their quit attempt with a free e-cigarette sooner. For some participants, however, the e-cigarette being free was not a key factor.

##### Subtheme 1B: Learning and Using the E-Cigarette

Participants felt they were given the right amount of information about the e-cigarette via the advisers. Participants reported varying opinions about how long the e-cigarettes lasted before needing to be disposed of or recharged, highlighting the role of individual usage patterns. One participant described needing but not having a spare coil for the refillable e-cigarette and so had to buy a new e-cigarette before their next appointment to handle cravings. Another participant raised concerns that the length of time e-cigarettes are supplied for may be too short and risk individuals not being able to maintain a quit attempt.

##### Subtheme 1C: Role of Behavioural Support

The behavioural support (e.g., face-to-face visits and encouragement) provided by the advisors at both sites was described as helpful, non-judgemental, and informative, with regular phone or text contact and in-person visits. The friendly and dedicated adviser approach was appreciated. Support not only addressed smoking behaviours but also the wider factors that can contribute to smoking including stress. Two participants summarised the benefits of combining the offer of an e-cigarette with behavioural support. 

##### Subtheme 1D: Overall Perceptions of the Pilot

Participants had predominantly positive experiences with the pilot, with many saying they would recommend it to others. A range of perspectives were reported regarding how accessible and timely the pilot was, with one participant describing a smooth process. However, another participant experienced delays in accessing Pilot 2, likely due to staffing issues. 

#### 4.2.2. Theme 2: Preferences for Using E-Cigarettes

This theme describes participants’ negative perceptions and experiences of traditional NRT (subtheme 2A) and the advantages of using an e-cigarette (subtheme 2B). 

##### Subtheme 2A: Negative Perceptions and Experiences of Traditional NRT

Some, but not all, participants had previously tried NRT products. However, most participants held generally negative perceptions of NRT due to factors including taste. Though one person found the patches to be good during a previous quit attempt, more participants described unpleasant physical sensations and symptoms during pregnancy. For these reasons, participants preferred e-cigarettes over other forms of NRT as an aid to stop smoking. 

##### Subtheme 2B: Advantages of Using E-Cigarettes

One key advantage of e-cigarettes over traditional NRT products was the behavioural similarity of using the e-cigarette compared with combustible cigarettes. They were also perceived as less harmful. However, not all participants found the e-cigarettes to be sufficiently similar. Several participants liked the control offered by the e-cigarette in terms of monitoring usage. Whilst some participants maintained a routine of going to use the e-cigarette outside, using the e-cigarette indoors was another benefit. One participant perceived this option could be particularly useful for someone experiencing pregnancy-related fatigue or nausea.

#### 4.2.3. Theme 3: Journeys to Quitting Tobacco

This theme captures participants’ motivations to quit tobacco (subtheme 3A), barriers to quitting (subtheme 3B), the impact of quitting cigarettes (subtheme 3C), and the effectiveness of the pilot for quitting during pregnancy, as well as future smoking intentions (subtheme 3D).

##### Subtheme 3A: Motivations to Quit

For all pregnant women, their pregnancy and the health of the baby were key motivators for quitting smoking. One participant emphasised that pregnancy had been the only factor motivating them enough to want to quit. Other sources of motivation were to save money or because of encouragement from a partner, family member, or health professional.

##### Subtheme 3B: Barriers to Quitting

Participants reported several barriers to quitting or factors that made avoiding cigarettes more challenging. Two participants described the length of time they had been smoking as a potential barrier. Seeing other people smoking was another challenge for motivation. Whilst cigarette smoke increased the desire to avoid smoking for some participants, for one participant cigarette smoke contributed to cravings. Several participants discussed their use of smoking as stress relief, highlighting stress as a barrier to quitting. Another participant highlighted the association between depression and motivation to quit. A further challenge was that participants’ partners were unable to access a free e-cigarette in Pilot 1 (whereas Pilot 2 did support where possible), which may otherwise have contributed to joint quitting attempts. 

##### Subtheme 3C: Impact of Quitting

Quitting cigarettes within the pilot had various impacts on participants’ health and behaviours. Some participants described benefits to mental wellbeing, including feelings of pride and confidence, after using an e-cigarette. Several participants had already noticed or expected improvements in physical health symptoms, such as reduced chest infections and wheezing. Two participants appreciated that their clothes and homes no longer smelt of cigarettes. The perceived impact on partner smoking behaviour was mixed. Sometimes partners continued smoking and others also tried to quit using e-cigarettes with mixed success. One participant described how having an e-cigarette around could influence their partner to sometimes use it rather than a combustible cigarette.

##### Subtheme 3D. Effectiveness of the Pilot for Quitting and Future Smoking Intentions 

Most participants reported that they had either quit cigarettes completely or had reduced their cigarette use. A few other participants described dual use throughout the pilot and others were more quickly able to eliminate cigarettes entirely. Some participants also found the regular carbon monoxide readings motivating and a measure of success.

There was a notable uncertainty around whether participants would be able to or wanted to quit smoking or e-cigarettes in the longer term. Several participants planned to continue smoking at a reduced level or to combine cigarettes with e-cigarettes. Some participants considered e-cigarette use as something they would keep doing. Other participants had stronger intentions to maintain smoking cessation.

## 5. Discussion

This study describes the experiences and perceptions among individuals who had accepted e-cigarettes as part of smoking cessation in pregnancy services in two UK sites and offers an important contribution to the evidence base given the paucity of qualitative insight in this area [25,29]. Overall, participants reported that e-cigarettes, in combination with behavioural support, were helpful for reducing smoking tobacco. Our findings broadly align with previous qualitative research from a multi-centre randomised controlled trial, where pregnant women positively perceived e-cigarettes to be necessary for smoking cessation and outweighed any concerns [30]. Though limited by low trial uptake, e-cigarettes were also indicated to be more effective for quitting than NRT patches [20]. There were largely favourable opinions of the free e-cigarette devices, though mixed successes with finding a preferred flavour. Previous research with a larger sample of pregnant women deduced overall preferences for sweet, fruit, and mint flavours over tobacco flavours [31]. Our evaluation highlights the importance of providing a range of flavours to suit individual preferences, as some individuals found replicating tobacco to be a useful strategy for maintaining motivation. Perceptions of the behavioural support (e.g., face-to-face visits and encouragement) were overwhelmingly positive and, along with providing adequate information and CO verifications, helped women’s motivation to quit within the pilot, consistent with wider understanding [32]. Participants particularly valued the supportive interactions from professionals, consistent with broader evidence and guidelines that non-judgemental support promotes motivation and behaviour change [32,33].

Participants expressed preferences for e-cigarettes over other NRT products and liked the behavioural similarities with smoking tobacco, although the e-cigarette did not satisfy cravings for everyone. The existing literature suggests a range of reasons for choosing e-cigarettes as a strategy towards this goal, including being able to use e-cigarettes in smoke-free areas and similar hand-to-mouth action [34,35]. Participants also described that the e-cigarette being free had enabled them or could enable others to engage in a quit attempt. This finding warrants wider exploration given that previous research has indicated lower effectiveness of typical smoking cessation interventions among pregnant women from lower socioeconomic backgrounds [13]. 

Research has already identified that pregnancy and the baby’s health are key motivating factors for smoking cessation [19,34] and in this study, the pregnancy and the health of the baby were key motivators to quit smoking. It is also necessary to understand and address the potential barriers to smoking cessation in pregnancy to increase success. In this study, the numerous barriers included the length of time someone had smoked, others in the house smoking, cigarettes forming part of daily routines, depression, and stress, consistent with previous research [36]. Some women in this study also raised concerns about relapses due to post-partum stress, highlighting the benefits of continued social and professional support for stress and mental wellbeing [37]. Positively, most participants reported quitting cigarettes or reducing cigarette use, with reported benefits to mental and physical health and reduced smells of smoke. However, there was uncertainty around whether quits could be sustained post-partum and longer term and whether participants would stop using e-cigarettes in the future. Previous reviews have reported that the efficacy of e-cigarettes as a smoking cessation tool may decrease following birth [34] and national guidance recommends continuing NRT provision after pregnancy if required to prevent relapse [27]. Further evaluation of the maintenance of smoking behaviours postpartum and the role that e-cigarettes may play is therefore required.

Whilst the pilot aimed to achieve smoking cessation for pregnant woman, there was an unclear impact of e-cigarettes on partners’ smoking behaviour. Reducing the smoking behaviours of partners could have additional health benefits via reduced smoke exposure, as well as offering social support and reduced smoking cues [38]. Some participants highlighted potential opportunities to support partners to quit smoking and further investigation is needed to better understand the role of partners in smoking cessation during pregnancy [39]. 

The findings can inform future e-cigarette pilots, such as embedding non-judgemental behavioural support, providing appropriate information about e-cigarettes, supplying multiple flavours to suit various preferences, ensuring staffing resources can facilitate a smooth enrolment process, and embedding or signposting to longer-term support for smoking cessation and wellbeing.

### Strengths and Limitations

Interviewing pregnant women who were provided e-cigarettes from two different pilot sites is a key strength of the evaluation and developed insight into the similarities and differences among participants’ experiences. However, uptake to interviews was challenging, with many pregnant women being uncontactable after obtaining consent or at the time of scheduled interviews. Those interviewed all reported a white ethnicity and wider experiences and perceptions among individuals from other ethnicities are missing from these findings. Data were not explicitly collected on smoking habits or quitting history. The two areas in this research varied in demographics with Pilot 1 and Pilot 2 having a 92.2% and 55.3% proportion of the population reporting as white, respectively. We did not interview women who declined to take part in the pilot and so the possible barriers to taking up the e-cigarette offer are not reported.

## 6. Conclusions

Interviews in this study illuminated how pregnant women taking part in an e-cigarette pilot had positive experiences of using e-cigarettes and expressed preferences for e-cigarettes over NRT. There was reported success with quitting combustible cigarettes by using e-cigarettes, further supporting how e-cigarettes are increasingly recognised as a harm reduction method for pregnant smokers in the UK. The inclusion of behavioural support was also valued and the e-cigarette being free may have improved access to this smoking cessation strategy for individuals with lower incomes. However, there were numerous barriers to quitting and staying quit and women were often uncertain about whether they would quit smoking or e-cigarette use post-partum and longer term. Further research exploring the views of pregnant women from more diverse backgrounds and those who decline e-cigarettes is warranted, as well as further consideration of the role and impact of partners who smoke in this timeframe.

## Figures and Tables

**Table 1:** An overview of the themes generated from the interviews.

Themes	Subthemes	Quotes to Support
Perceptions of key aspects of the e-cigarette pilot	1A: The e-cigarette offered	“*it’s quite difficult to transport if it’s quite full because it may leak*” (P2a) “*I found that it goes green and lights up when you draw in […] if I was trying not to be seen smoking, I felt like I was a walking glow stick* (P1b) “*I personally preferred the tobacco one…It felt like you were smoking a cigarette because of the taste*” (P1e) “*if there was some way of you being able to...like test the, the vape juice…it tastes differently to what it smells*” (P2d) “*you’re not losing anything if it doesn’t work... with it being free, it’ll encourage a lot of people on low income to try it*” (P2c) “*I could afford to buy my own, it wasn’t necessarily about that for me, but yeah it was that kind of 1:1 support that I think just really did it for me*” (P1f)
	1B: Learning and using the e-cigarette	“*sometimes you can get information… it’s like woah it’s a little bit too much, but it didn’t feel like that*” (P2e) “*at first I was using one [e-cigarette] every other day … but once I started settling down and I was getting over that initial like wanting a cigarette all the time they last about four to five days*” (P1d) “*that’s the downside of the programme, because they need a spare coil from the beginning*” *(*P2a) “*I think the [e-cigarette] liquids should be supplied a little bit longer… what happens in 12 weeks’ time that I run out and I can’t afford to buy it?*” (P2f)
	1C: Role of behavioural support	“*she’s [adviser] been really supportive for me and she’s really tried*” (P2f) “[*adviser] said about you need to learn how to deal with your stress differently … it helped cut that down and re-evaluate how I handled stress”* (P1b) “*I think the vape plus all the extra support, it just makes it all worthwhile*” (P2e)
	1D: Overall perceptions of the pilot	“*my midwife, put me in touch… and then [adviser] text me and that was it. She was round in about four days*” (P2d) “*the only negative would have been how long it took for them [stop smoking in pregnancy service] to get in touch with me. I think, probably because it’s just the two of them doing it*” (P2c)
Preferences for using e-cigarettes	2A: Negative perceptions and experiences of traditional NRT	“*I got the gum, it doesn’t taste very nice*” (P2c) and perceived effectiveness: “*because it’d [patch] be on my arm…I feel like I’d forget a little bit and think I’d still maybe reaching for a fag [cigarette]*” (P2e) “*[patches] make a lot of people feel sick. I suffered with my sickness throughout my whole pregnancy. She [adviser] didn’t want to give me anything that was going to make me feel worse*” (P1e)
	2B: Advantages of using an e-cigarette	“*patches aren’t like cigarettes, gum isn’t like cigarettes, the nozzle things, they’re not like cigarettes. E-cigarettes are the closest you can get to a normal cigarette*” (P1c) “*[e-cigarette] still didn’t feel like a cigarette, so I still didn’t feel satisfied like if I had had a cigarette*” (P1h) “*you control it more. How much you vape… the amount of nicotine you are using*” (P2a) “*a lot of people who have morning sickness*…*you can actually sit just on your bed and relax and feel better, and have the nicotine [e-cigarette] quite close to you*” (P2a)
Journeys to quitting tobacco	3A: Motivations to quit	“*being pregnant was the only thing that’s ever really made me wanna quit smoking*” (P2c)
	3B: Barriers to quitting	“*when you’ve been doing it [smoking] for so many years you’re just in that kind of routine”* (P1c) “*it is very difficult to quit smoking when you live with someone that smokes. Because obviously the smell it, it lingers*” (P2c) “*because I’ve got depression, I’m not in a motivated state quite a lot of the time […] so my motivation goes down and then smoking goes up*” (P1c) “*my partner was still smoking…I said like isn’t there like something my partner can join on as well to kind of get the vape too so we could have done it together*” (P2f)
	3C: Impact of quitting	“*[using an e-cigarette] really makes me feel positive, and kind of makes my mind feel good*” (P2e) “*I don’t want to be running round in a play area or park and suddenly cough as I’m running and a smoker and can’t breathe so I feel healthier [using an e-cigarette]*” (P1b) “*when I tried it [e-cigarette], he tried it as well. He tried to, but I don’t think he stuck to it*” (P2c) “*he’s quite a heavy smoker […] sometimes he’ll say could I just have a little bit of your vape please? […] We’re actually gonna buy him one*” (P2d)
	3D: Effectiveness of the pilot for quitting and future smoking intentions	“*I was combining smoking with vaping just for a week or two…I was fully on vaping, almost straight away*” (P2a) “*when it [the carbon monoxide reading] was coming back as nothing … it just gave you that sense of achievement, you’re like oh wow!*” (P1d) “*I will probably keep using the vape...I think I will probably continue to use both [cigarettes and e-cigarette]. However, if I did choose to quit, that would be my method of going forward to quit*” (P2c) “*I can’t see me going back to the cigarettes. I would like to say that I’d completely stop vaping, but I can’t see that happening anytime soon. Because I don’t wanna rush off it too quickly and end up going back to smoking again*” (P2d) “*I don’t think I want to go back to smoking, no. I’ve done a massive achievement of not doing it for that long. I don’t see why I should go back to it*” (P1a)

## Data Availability

All data generated or analysed during this study are included in this published article.
